# Telomerase Induction in HPV Infection and Oncogenesis

**DOI:** 10.3390/v9070180

**Published:** 2017-07-10

**Authors:** Rachel Katzenellenbogen

**Affiliations:** 1 Center for Global Infectious Disease Research, Seattle Children’s Research Institute, Seattle, WA 98101, USA; rkatzen@uw.edu; Tel.: +1-206-884-1082; 2 Department of Pediatrics, Division of Adolescent Medicine, University of Washington, Seattle, WA 98195, USA

**Keywords:** human papillomavirus, papillomaviruses, oncogenic virus, cancer, HPV E6, HPV E7, hTERT, telomerase

## Abstract

Telomerase extends the repetitive DNA at the ends of linear chromosomes, and it is normally active in stem cells. When expressed in somatic diploid cells, it can lead to cellular immortalization. Human papillomaviruses (HPVs) are associated with and high-risk for cancer activate telomerase through the catalytic subunit of telomerase, human telomerase reverse transcriptase (hTERT). The expression of hTERT is affected by both high-risk HPVs, E6 and E7. Seminal studies over the last two decades have identified the transcriptional, epigenetic, and post-transcriptional roles high-risk E6 and E7 have in telomerase induction. This review will summarize these findings during infection and highlight the importance of telomerase activation as an oncogenic pathway in HPV-associated cancer development and progression.

## 1. Introduction: Human Papillomavirus Infections

### Human Papillomavirus Infection and Life Cycle in Stratified Squamous Epithelium

Human papillomaviruses (HPVs) are small, non-envelope, double stranded DNA viruses, and there are more than 200 papillomavirus types identified to date (see curated list at Papillomavirus Episteme (PaVE); https://pave.niaid.nih.gov/#home). All HPVs complete their life cycle in stratified squamous epithelium, either cutaneous or mucosal dependent on their tropism (reviewed in [1,2]). The HPV life cycle begins with infection of basal cells in stratified squamous epithelium. These cells are reached either through microabrasions or at anatomic sites where monolayer, columnar epithelium transition to stratified squamous epithelium. These sites of transition are the found at the cervical transformation zone, the anal verge, and crypts in the oral mucosa, and intriguingly these are also where many HPV-associated cancers occur [3]. There is also evidence the cervix and anal verge contain specific cells that support HPV-associated cancers and have signature gene expression profiles [4,5]. Whether or not these cells represent a preferred host for the virus or more narrowly for the initiation of cancer, we broadly understand that a productive HPV infection begins in the bottom layer of stratified squamous epithelium.

In the basal layer, the HPV genome escapes its viral capsid and is maintained at 50–60 copies per cells (ranging from 10 to 200 copies) [2,6], while the early (*E*) viral genes are expressed. E1 and E2 support HPV DNA replication and the measured expression of E6 and E7 [2,6]. One function of E6 and E7, as well as E5, is to reduce activation of the innate immune system and avoid viral clearance [7,8,9]. Evading immune sensing and maintaining copies of the viral genome are both critical to establishing an infection.

As cells leave the basal layer, they rise through the suprabasal and spinous layers and progress through differentiation. HPV requires this cellular differentiation program to complete its own viral life cycle. Without it, HPV has an abortive infection. Therefore, as HPV-infected host cells rise through the differentiating layers, HPV progresses through its own life cycle. In these differentiating layers, HPV DNA becomes amplified to several thousand copies per cell [2], and late (*L*) gene expression is activated. L1 and L2 form the protein shell of HPV, and they incorporate the HPV episomal DNA into new infectious virions. Those released as epithelial cells are sloughed off at the top of stratified squamous epithelium, completing the viral life cycle.

Although HPV requires its host cell to differentiate in order to complete its life cycle, it also requires it host cell to continue to grow when normally it would not. HPV dysregulates the balance of growth and differentiation found in stratified squamous epithelium to do so. In low-risk HPV types, this is manifest as warts. In high-risk HPV types, this leads to dysplasias and carcinoma in situ [10]. This dysregulation of growth and differentiation is driven primarily by the *E6* and *E7* genes. E6 and E7 drive cells to continue to grow and divide when they otherwise would not, and, to that end, E6 and E7 are expressed in the differentiating layers of stratified squamous epithelium [2,10]. By E6 and E7 disrupting the typical segregation of cell cycle and growth from differentiation, more HPV DNA can be copied and expressed, and more cells infected by HPV can grow.

There are at least 15 HPV types that are defined as high-risk (HR) by their association with cervical cancer [11]. HPV-associated cancers universally express the HR *E6* and *E7* genes, thus are considered to be HPV’s viral oncogenes. If HR E6 and E7 are introduced into normal diploid cells, they become immortalized [12,13]. If HR E6 and E7 expression is reduced in HPV-positive cervical cancer cell lines, the cells growth arrest [14,15]. This implies that not only are the HR *E6* and *E7* genes required for oncogenesis, but they are also required for the maintenance of malignant phenotype. There are critical oncogenic pathways that HR E6 and E7 affect. HR E7 targets the retinoblastoma protein (Rb) for degradation in epithelial cells [16,17]. This allows infected epithelial cells to proceed through S phase and the cell cycle. HR E6 targets p53 for degradation to avoid apoptosis [18,19]. It similarly targets PSD-Dlg-ZO-1/2 (PDZ) containing proteins for degradation, disrupting cellular apicobasal orientation and cell-to-cell adhesion in the epithelium, leading to hyperplasia [20,21,22,23,24]. HR E6 also activates gene expression; its most critical gene to activate is human telomerase reverse transcriptase (hTERT), the catalytic subunit of telomerase. It is the degradation of Rb by HR E7 and the activation of hTERT by HR E6 that drives normal keratinocytes to immortalization [12,13].

In this review article, we will describe the roles E6 and E7 have in telomerase induction during HPV infection and in oncogenesis. We will first define telomeric DNA, its role in DNA protection, and the enzymatic function of telomerase. Then, we will highlight the multiple ways HR E6 and E7 derepress hTERT to activate and accelerate telomerase activity. Finally, we will discuss how E6, E7, and hTERT expression changes during oncogenesis.

## 2. Telomeric DNA and Telomerase

Telomeric DNA caps the ends of linear chromosomes, is repetitive, and is approximately 5000 to 15,000 nucleotides in length in humans [25,26]. No genetic material is found within telomeric DNA itself. Rather, it is bound by the shelterin protein complex to block double strand (dsDNA) repair signaling [27], protecting against non-homologous end joining and erroneous chromosomal break repair [27]. Telomerase, a ribonucleoprotein enzyme complex, extends this repetitive telomeric DNA. The holoenzyme includes the catalytic subunit hTERT that is expressed at rate-determining levels [28,29,30], the telomerase RNA component, TERC or TR, used to extend the six nucleotide repeat 5′ TTAGGG 3′ found in telomeric DNA, and the protein dyskerin [26,31]. Telomerase is typically active during embryonic and fetal development [32] and in stem cells [33]. It is not active in normal somatic cells. However, telomerase activity has been detected in almost all human tumors and immortalized cells in culture [29,30,34].

Without telomerase activity, the linear chromosomes of cellular DNA are serially shortened with every cell cycle and division by 100 to 200 nucleotides [35]. This DNA loss is called the “end replication problem”. As telomeric DNA becomes critically shortened over time, normal somatic diploid cells enter mortality stage one (M1) and undergo either replicative senescence or apoptosis [35,36,37,38]. If these cells continue to cycle beyond stage M1, they lose the protective shelterin protein complexes and enter mortality stage two (M2) or crisis. In crisis, cells signal that there are dsDNA breaks at the ends of chromosomes requiring repair. This genomic instability creates the “end protection problem”. It leads to anaphase bridges and chromosomal breaks that are catastrophic to the cell [39,40,41]. Only clonal cells survive that have had enormous chromosomal rearrangements [41]. Consequently, the extension of telomeric DNA by telomerase allows diploid cells to grow over time, avoiding apoptosis, senescence, and chromosomal rearrangements. It is because of this allowance that telomerase and its rate-determining catalytic subunit hTERT are expressed in nearly all cancers [30,34,42,43].

## 3. Telomerase and hTERT Activity Driven by HR E6 and E7

Studies in the late 1980s defined the roles HR E6 and E7 played in cellular immortalization, cancer development, and cancer progression [12,13]. In a seminal paper by Kiyono et al. HR E6 and E7 were found to collaborate in the immortalization of both primary fibroblasts and keratinocytes, specifically by dysregulating Rb/p16INK4A and telomerase [44]. HR E7 was important for immortalization, but it did not directly affect telomerase [44]. Rather, HR E6 did. HR E7 was described to increase hTERT driven expression of luciferase and augment telomerase activity driven by HR HPV E6 [45]. In HeLa cells, re-expression of either HR E6 or E7 after their removal led to increased hTERT [46]. Although E7 could synergize the E6 regulation of telomerase and cellular immortalization, HR E6 is the principal trigger and regulator of hTERT expression and telomerase activity (Table 1). Other studies built on these foundational reports.

Recent studies have confirmed that low-risk (LR) E6 does not activate telomerase [47] while HR E6 is necessary and sufficient for telomerase activation in keratinocytes [48,49,50,51]. Without HR E6, telomerase is not detected in epithelial cells, and the catalytic subunit of telomerase, hTERT, is not expressed [48,50]. In addition to HR E6 regulating the activity of telomerase, HR E6 was found to bind hTERT itself and repetitive telomeric DNA [52]. Therefore, the role HR E6 has in hTERT, telomerase, and telomeric DNA regulation is multilayered, demonstrated by its redundant actions to drive immortalization. 

The E3 Ubiquitin Ligase E6 Associated Protein (E6AP) is important for the activation of hTERT expression and telomerase activity by HR E6 [53,54,55]. E6AP partners with HR E6 to polyubiquitinate and degrade p53 and PDZ-containing proteins [18,19,21,56,57], but this partnership does not lead to the degradation of hTERT or telomerase; instead, it increases hTERT and telomerase. Decreasing HR E6 and E6AP by microRNA (miR375) indirectly reduced hTERT and telomerase activity in cells [58], and the E6 motifs needed to bind E6AP were also required for telomerase activation and immortalization in fibroblasts and keratinocytes [59]. Hence, HR E6 and E6AP (E6/E6AP) function as principal inductors of telomerase, and its catalytic subunit hTERT.

### 3.1. hTERT: Promoter Regulation

Most research on telomerase regulation has focused on the expression of its catalytic subunit, hTERT. The *hTERT* gene is constitutively repressed in somatic epithelial cells; this repression occurs at its promoter. The hTERT promoter is approximately 1100 nucleotides in length, with its core promoter being only 200 to 300 nucleotides long [60,61,62]. Normally, transcriptional repressors of hTERT are bound to *cis* elements in its core promoter, blocking transcription [48,60,61,63,64,65,66,67,68,69]. These *cis* elements are E boxes, GC-rich sites, and X boxes.

Two E box *cis* elements flank the transcriptional start site of hTERT [61,62,66], and if these E boxes are mutated or deleted, hTERT expression and telomerase activity are dramatically reduced [50,54]. These E boxes are normally bound by c-Myc as a heterodimer with either Max or Mad. These c-Myc/Max or c-Myc/Mad heterodimers are important for hTERT transcriptional activation or repression, respectively [65,66,70,71,72]. Upstream transcription factor 1 (USF1) also binds to E boxes, competitively and sterically repressing hTERT expression by c-Myc/Max [50,73,74] Although the amount of c-Myc that binds to the hTERT promoter does not correlate to hTERT expression, the presence of c-Myc at the promoter is important [51,72,75]. E6/E6AP are also bound at E boxes in the hTERT promoter [51,54,74,75], and they interact with c-Myc to drive gene expression [51]. The requirement for E6AP to drive hTERT expression at the promoter with HR E6 is controversial [53,54,55,76,77], but the *cis* E boxes within the hTERT promoter are required for its transcriptional activation with or without HR E6.

There are five GC-rich *cis* elements in the hTERT promoter 5′ of the transcriptional start site [50,71,74]. Sp1 binds to these elements and transcriptionally activates hTERT expression [50,71]. Maz also is bound at these sites but is a transcriptional repressor [71]. Like deletion of the E boxes, deletion of GC-rich *cis* elements leads to loss of hTERT promoter-driven transcriptional activation [50].

Finally, there are two X boxes in the hTERT promoter [48]. One is downstream of the hTERT transcriptional start site, lies within the 5′ UTR of hTERT, and overlaps with the downstream E box to which c-Myc/Max binds [48]. The second is upstream of the hTERT core promoter in an inverted position [48]. Nuclear transcription factor X-box binding 1, isoform 3 (NFX1-91) is a repressor of hTERT transcription and is bound constitutively at the hTERT downstream X box [48,78]. NFX1-91 is polyubiquitinated by E6/E6AP and targeted for proteasomal degradation [48]. Its removal from the hTERT promoter leads to transcriptional activation of hTERT [48].

### 3.2. hTERT: Epigenetic Regulation

Beyond studies of the hTERT promoter *cis* elements and the transcriptional proteins that bind those elements, epigenetic studies of the hTERT promoter demonstrate important structural chromatin changes that affect transcriptional activation of hTERT [78]. Several studies document the importance of E6/E6AP in opening the hTERT promoter chromatin structure as they change histone acetyltransferase (HAT) and histone deacetylase (HDAC) recruitment to the hTERT promoter [53,78]. The hTERT repressor NFX1-91 not only binds the promoter X box *cis* element but also binds SIN3 transcription regulator family member A (mSin3A), a transcriptional co-repressor that recruits HDACs to promoters [78]. When NFX1-91 is degraded by E6/E6AP, HDAC activity at the hTERT promoter is lost and HAT activity increases [78], and with this, histone acetylation increases further over time [53].

DNA methylation patterns at the hTERT promoter also shift during an HPV infection and in tissue culture studies of HPV positive cells. Specific regions of the promoter become hypermethylated, while other regions become hypomethylated, during long-term tissue culture of cells with HR E6 and E7 [79,80,81,82]. Although a direct causal association between hTERT promoter methylation and cancer development has not been seen [83], there are changes that parallel increases in hTERT expression, and these changes in methylation patterns correlate with HR and probable HR E6 expression [84].

### 3.3. hTERT: Post-Transcriptional Regulation

Post-transcriptional regulation of hTERT by alternative mRNA splicing and mRNA stabilization is important for telomerase activity [32,85,86]. In non-HPV studies, c-Myc shifts hTERT mRNA expression from a non-active splice variant to an active form [87]. RNA processing proteins, such as Serine-Arginine Rich Splicing Factors, are also expressed at increased levels in high-grade cervical dysplasias [88], pointing indirectly to HR HPV manipulating RNA processing proteins during oncogenesis.

We found that hTERT and telomerase activity are upregulated post-transcriptionally by HR E6 through the host cellular protein NFX1-123 [86]. NFX1-123 is the longer splice variant of the *NFX1* gene (the hTERT transcriptional repressor NFX1-91 is the shorter splice variant) [48,89]. Greater expression of NFX1-123 leads to increased hTERT and telomerase activity with HR E6, and knock down of endogenous NFX1-123 reduces the ability of HR E6 to increase hTERT and telomerase [86,89]. The mechanism by which NFX1-123 augments hTERT expression is through stabilization of the hTERT mRNA, and the 5′ UTR of the hTERT transcript is necessary for this stabilization [86].

NFX1-123 contains two protein motifs important for binding, stabilizing, and augmenting hTERT expression. The R3H domain of NFX1-123 has putative single-stranded nucleic acid binding capabilities [86,89,90], and when this motif is deleted, the stabilization and increased expression of hTERT seen in HR E6 expressing cells is lost [86,89]. Second, the poly(A) binding protein interacting motif (PAM2) of NFX1-123 directs binding of cytoplasmic poly(A) binding proteins (PABPCs) to NFX1-123, and PABPCs increase the stability and translation of genes with poly (A) tailed mRNA [89,91]. Like the R3H domain, when the PAM2 motif of NFX1-123 is mutated or deleted, its ability to augment hTERT expression and telomerase activity by HR E6 is also lost [86,89].

Cytoplasmic poly(A) binding proteins themselves are important in hTERT expression and telomerase activity in HR E6 positive cells. When PABPC types 1 and 4 are knocked down, hTERT and telomerase activity are reduced [92]. Conversely, when PABPC type 4 is overexpressed, hTERT and telomerase are augmented, and cells with either more hTERT or more PABPC type 4 grow better in culture [92].

Collectively, these research findings highlight multiple ways hTERT mRNA is post-transcriptionally regulated. Again, the duplicative mechanisms, from DNA, chromatin, and RNA regulation, that HR E6 uses to increase hTERT and telomerase emphasizes its importance to HPV and oncogenesis.

### 3.4. hTERT: Beta HPV E6

Most studies examining the regulation of telomerase by HPV have focused on HR HPVs from the α genus. More recent work has examined the role β genus HPVs play in nonmelanomatous squamous cell carcinoma, and how the beta E6 and E7 proteins may also activate oncogenic pathways, whether similar or disparate to α HR E6 and E7 proteins. Work by Galloway et al. has determined the oncogenic potential of β HPV types through direct analysis of their E6 and E7 protein functionality, and specifically how different E6 types activate hTERT expression, telomerase activity, and immortalization [40,93]. β E6 proteins with greater effect on hTERT activation and telomerase activity have improved cellular growth and longevity in culture [93]. This improvement is not only proportional to telomerase activity but also depends on the presence of E6AP [93]. Therefore, like α HR HPV types, several β genus *E6* genes drive hTERT expression and telomerase activity.

## 4. Telomerase in HPV-Associated Cancers

**Figure 1:**
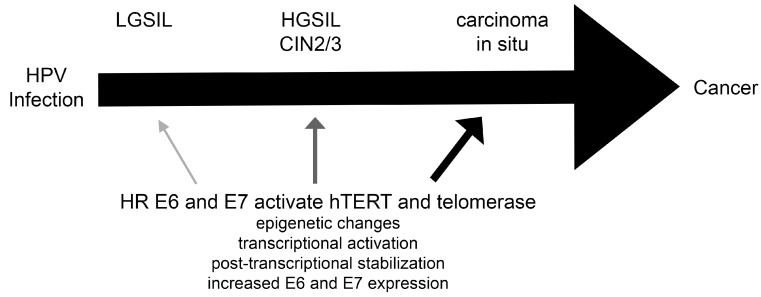
HPV infection and telomerase induction. Telomerase, and its rate determining catalytic subunit, hTERT, is normally not expressed in somatic cells. With a HR HPV infection, E6 and E7 activate the *hTERT* gene. With disease progression, *hTERT* activation and telomerase activity increases (demonstrated by darker, larger arrows), and the expression of HR E6 and E7 also increases with the integration of HPV DNA into the host cell chromosomal DNA or loss of E2 regulation. LGSIL (low-grade squamous intraepithelial lesion) is typical for an active HPV infection. HGSIL (high-grade squamous intraepithelial lesion) is typical for a HR HPV infection with worsening cytologic changes and parallel greater histologic involvement, with multiple layers of the stratified squamous epithelium. CIN2/3 (cervical intraepithelial neoplasia 2 or 3) shows histologic changes due to an active HPV infection that involved most (2) or all (3) of the stratified squamous epithelium. Carcinoma in situ is the full thickness involvement of stratified squamous epithelium without breakdown of the basement membrane.

During cervical cancer initiation and progression, the expression of hTERT and the activity of telomerase parallels worsening disease [94,95,96,97]. Approximately half of HPV positive squamous intraepithelial lesions and cervical intraepithelial grade III lesions have detectable telomerase activity and that increases to over 90% in HPV positive cervical cancer samples [94,98]. The level of hTERT expression and telomerase activity found in cervical lesions is proportional to the pathologic severity of disease detected [94,96,98]. In HPV-positive cancers, telomerase is universally expressed (modeled in Figure 1) [34]. Telomerase is increasingly identified as having both canonical and non-canonical functions, and each is important to HPV-induced cellular immortalization and oncogenesis [99].

Interestingly, during the transition from HR HPV infection, to dysplasia, to frank cancer, HPV DNA typically no longer remains episomal. It becomes integrated in the cell’s chromosomes. This happens within the context of genomic instability, created and supported by the functions of HR E6 and E7 themselves. This, by definition, means HPV can no longer form infectious virions; it also means HPV gene expression itself is dysregulated. With HPV DNA integration, the HR E6 and E7 genes are universally preserved, but the regulatory *E1* and *E2* genes are often lost. Even in non-integrated HPV driven cancers, the binding sites for E2 often become methylated. These changes allow for greater expression of E6 and E7, as E2 moderates the expression level of these viral oncogenes [100,101]. Although not required, with increased E6 and E7, there is a parallel increase in telomerase activity [102]. During HR HPV infection and its associated cancer development and progression, HR E6, with E7, activates telomerase. This activation is augmented over time by changes that support cellular immortalization and growth and by the acceleration of viral and cellular genomic instability that was first initiated by HR E6 and E7.

High-risk HPV infections are associated with cancers in other sites besides the cervix [3]. These include vulvar, vaginal, anal, penile, and the head-and-neck. Each of these HPV-associated cancers are also associated with upregulated telomerase activity [34]. Therefore, the anatomic location of a HR HPV infection is not the singular instigator of immortalization—the commonality among these cancers is HR HPV, and HR E6 specifically, driving telomerase.

## 5. Conclusions

High-risk E6 hijacks host cell proteins from their usual function (E6AP, c-Myc, HDAC, HAT, mSin3A, NFX1-91, NFX1-123, PABPCs, and mRNA splicing factors) to activate hTERT and telomerase activity. This supports cellular immortalization. These viral-cellular protein partnerships primarily control the derepression of telomerase’s catalytic subunit, hTERT. They increase hTERT through the promoter’s *cis* and *trans* elements, the chromatin structure, the mRNA product, and associated RNA regulatory proteins. There are still many unanswered questions in the dysregulation of telomerase activation by HPV during infection and oncogenesis. However, its universality implies it is critical to the core function of HR HPV types and to induction of cancers caused by HPV.

## Figures and Tables

**Table 1:** HR E6 and E7 regulation of hTERT and cellular protein targets for that regulation.

HPV Gene	Effect on hTERT	Cellular Protein Target
**Chromatin Effects**		
*E6* and *E7*	Promoter methylation changes	
*E6*	Increase promoter acetylation	HATs and HDACs, mSin3A
**Transcription Effects**		
*E6*	Increase transcriptional activators	c-Myc/Max, Sp1
*E6*	Decrease transcriptional repressors	c-Myc/Mad, Maz, USF1, NFX1-91
*E7*	Increase expression with E6	
**RNA Effects**		
*E6*	Increase transcript stability	NFX1-123, PABPCs
*E6*	Increase active spliced isoform of hTERT	c-Myc
**Protein Effects**		
*E6*	Binds hTERT	hTERT

HR: high-risk; hTERT: human telomerase reverse transcriptase; HPV: human papillomavirus; HATs: histone acetyltransferases; HDACs: histone deacetylases; mSin3A: SIN3 transcription regulator family member A; c-Myc: MYC proto-oncogene; Max: MYC associated factor X; Maz: MYC associated zinc finger protein; USF1: Upstream transcription factor 1; NFX1-91: Nuclear transcription factor, X-box binding 1, isoform 3; NFX1-123: Nuclear transcription factor, X-box binding 1, isoform 1; PABPCs: cytoplasmic poly(A) binding proteins.
